# *De novo* Transcriptome Assembly of *Senna occidentalis* Sheds Light on the Anthraquinone Biosynthesis Pathway

**DOI:** 10.3389/fpls.2021.773553

**Published:** 2022-01-03

**Authors:** Sang-Ho Kang, Woo-Haeng Lee, Joon-Soo Sim, Niha Thaku, Saemin Chang, Jong-Pil Hong, Tae-Jin Oh

**Affiliations:** ^1^Genomics Division, National Institute of Agricultural Sciences, RDA, Jeonju, South Korea; ^2^Department of Life Science and Biochemical Engineering, SunMoon University, Asan, South Korea; ^3^Metabolic Engineering Division, National Institute of Agricultural Sciences, RDA, Jeonju, South Korea; ^4^Genome-Based BioIT Convergence Institute, Asan, South Korea; ^5^Department of Pharmaceutical Engineering and Biotechnology, SunMoon University, Asan, South Korea

**Keywords:** *Senna occidentalis*, anthraquinone, secondary metabolite, transcriptome analysis, transcription factor

## Abstract

*Senna occidentalis* is an annual leguminous herb that is rich in anthraquinones, which have various pharmacological activities. However, little is known about the genetics of *S. occidentalis*, particularly its anthraquinone biosynthesis pathway. To broaden our understanding of the key genes and regulatory mechanisms involved in the anthraquinone biosynthesis pathway, we used short RNA sequencing (RNA-Seq) and long-read isoform sequencing (Iso-Seq) to perform a spatial and temporal transcriptomic analysis of *S. occidentalis*. This generated 121,592 RNA-Seq unigenes and 38,440 Iso-Seq unigenes. Comprehensive functional annotation and classification of these datasets using public databases identified unigene sequences related to major secondary metabolite biosynthesis pathways and critical transcription factor families (bHLH, WRKY, MYB, and bZIP). A tissue-specific differential expression analysis of *S. occidentalis* and measurement of the amount of anthraquinones revealed that anthraquinone accumulation was related to the gene expression levels in the different tissues. In addition, the amounts and types of anthraquinones produced differ between *S. occidentalis* and *S. tora*. In conclusion, these results provide a broader understanding of the anthraquinone metabolic pathway in *S. occidentalis*.

## Introduction

*Senna occidentalis* (L.) Link (subfamily, Caesalpinioideae; family, Leguminosae; also known as *Cassia occidentalis* L.) is a toxic leguminous annual plant widely distributed across tropical and subtropical regions in Europe, Australia, the southern and eastern United States, South Africa, South Asia, and India (Tokania et al., 2000). It is also found in a variety of environments, including roadsides, pastures, forests, coastal environs, and crop fields (Vashishtha et al., 2009; Pal et al., 2019). *S. occidentalis* is widely consumed in various countries and harvested from the wild for various pharmacological purposes, such as the treatment of hypertension, diabetes, dropsy, biliousness, fever, ringworm, eczema, rheumatism, eye pain, hematuria, typhoid fever, and tuberculosis (Burkill, 1995; Gidado et al., 2016). *S. occidentalis* contains polyphenols and anthraquinone derivatives that exhibit a variety of pharmacological properties, including antibacterial, antifungal, antioxidant, antiplasmodial, and antiparasitic activities (Yen and Chen, 1995; Bakhiet and Adam, 1996; Luximon-Ramma et al., 2002; Chukwujekwu et al., 2005; Duraipandiyan and Ignacimuthu, 2007; Morita et al., 2007).

In addition to phytochemicals such as kaempferol, sitosterol, tannin, and xanthine, *S. occidentalis* also produces anthraquinone derivatives, such as aloe-emodin, chrysophanol, obtusin, and physcion (Kudav and Kulkarni, 1974; Chukwujekwu et al., 2005; Verma et al., 2010; Li and Li, 2017). Anthraquinones have been used to treat various diseases and possess strong pharmacological activities, such as laxative, anti-cancer, anti-bacterial, anti-inflammatory, anti-injury, and antioxidant properties (Jung et al., 2016). About 700 anthraquinone derivatives have been reported, of which 200 are present in plants (Duval et al., 2016). Anthraquinones are generally classified as anthraquinone monomers, which can be subdivided into hydroxy anthraquinones, anthranones, anthranols, and bianthraquinones, depending on the structure of the parental nucleus (Zhao et al., 2016). The phenolic hydroxyl group of the anthracene nucleus has a great influence on its anti-cancer properties (Li and Jiang, 2018). In addition, anthraquinone monomers such as emodin, aloe-emodin, chrysophanol, and physcion have various physiological activities that might enable them to be used in treatments targeting cancer, viruses, and depression.

The secondary metabolites of an organism, such as anthraquinone derivatives, are produced through a variety of precursors and biosynthesis pathways. In particular, the most important gatekeepers in the biosynthesis of secondary metabolites are the enzymes that biosynthesize the backbones of the metabolites. The type III polyketide synthase (PKS) enzymes are found in bacteria, fungi, and plants (Austin and Noel, 2003; Austin et al., 2004; Funa et al., 2005; Seshime et al., 2005) and specialize in the production of important secondary metabolites (aromatic polyketides), which have potential nutraceutical, cosmeceutical, and pharmaceutical applications of increasing economic interest (Lauritano et al., 2017; Martínez et al., 2019). This enzyme has two independent active sites that catalyze a series of decarboxylation, condensation, and cyclization reactions (Tropf et al., 1995). The type III PKSs include chalcone synthase (CHS), stilbene synthase (STS), and acridone synthase (Dao et al., 2011), all of which have already been extensively studied in several plant species. One of the products of polyketide synthase, anthraquinone, is produced by bacteria, fungi, insects, and plants (Chiang et al., 2010; Shamim et al., 2014). Through several studies, two biosynthesis pathways for anthraquinone have now been proposed: the chorismite/*O*-succinyl benzoic acid pathway and the polyketide pathway (Burnett and Thomson, 1968; Bauch and Leistner, 1978). The former pathway was observed in the Rubiaceae plant family, while the latter is found in bacteria, fungi, and insects (Awakawa et al., 2009; Chiang et al., 2010).

Studies of anthraquinone biosynthesis in *Senna tora* have provided some information on secondary metabolites in the *Senna* genus. In a recent study, the anthraquinone scaffolds atrochrysone carboxylic acid and endocrocin anthrone were found in *S. tora*; these scaffolds are generated by the activity of chalcone synthase-like (CHS-L) enzymes (Kang et al., 2020b). Kang et al. identified 28 type III PKSs, among which 16 genes similar to *CHS-L* were systematically classified. Interestingly, the *CHS-L* gene group was only expanded in some species, in which it was identified as a separate gene subfamily, but was not expanded in another 11 species. The *in vitro* analysis of one CHS-L enzyme (CHS-L9) resulted in the generation of atrochrysone carboxylic acid and endocrocin anthrone, which are believed to be the backbone of anthraquinone, which confirmed that this enzyme is associated with the anthraquinone biosynthesis pathway in *S. tora* (Kang et al., 2020b).

This study aims to provide insights into the anthraquinone biosynthesis pathway through the transcriptomic analysis of *S. occidentalis*, which belongs to the same genus as *S. tora*. In our previous study, we presented the *de novo* transcriptome of *S. tora*, elucidating its anthraquinone biosynthesis pathway. Here, we reveal clear differences between *S. occidentalis* and *S. tora* through the transcriptomic and metabolomic analysis of *S. occidentalis*. Furthermore, we explore the variation in anthraquinone production in the different developmental stages and tissue types in these plants.

## Materials and Methods

### Plant Materials and RNA Preparation

For the transcriptomic analysis, specimens of *S. occidentalis* (voucher number: IT 175099 from the National Agrobiodiversity Center, South Korea) grown in the field at the National Institute of Horticultural and Herbal Medicine (Eumseong, South Korea) were used. Leaf, root, and young and mature seed tissues were harvested from healthy plants and stored at −80°C until use for RNA extraction.

Total RNA was extracted from the leaf, root, and seed tissues using an RNeasy Plant Mini kit (Qiagen, Hilden, Germany). The RNA purity was measured using a NanoDrop 8000 spectrophotometer (Thermo Fisher Scientific, Waltham, MA, United States) and a 2100 Bioanalyzer (Agilent Technologies, Santa Clara, CA, United States). Only samples with a minimum total RNA integrity value of 7 were used.

### Illumina Short-Read Sequencing

Poly(A)^+^ mRNA was twice purified from 1 μg total RNA using poly(T) oligo-attached magnetic beads and then fragmented. The randomly cleaved RNA fragments were reverse-transcribed into first-strand cDNA using reverse transcriptase, random hexamer primers, and dUTP. A single A base was added to these cDNA fragments, after which an adapter ligation was performed. The product was purified and enriched using polymerase chain reaction (PCR) to create the final-strand cDNA library. The quality of the amplified library was confirmed using capillary electrophoresis (bioanalyzer; Agilent Technologies). Quantitative PCR (qPCR) was conducted using SYBR Green PCR Master Mix (Thermo Fisher Scientific), after which equal amounts of the indexed tagged libraries were aggregated. The cBot-automated cluster creation system (Illumina, San Diego, CA, United States) was used to perform cluster generation in a flow cell. Sequencing was performed with a reading length of 2 × 100 base pairs (bp) by loading the flow cell into the HiSeq 2500 sequencing system (Illumina). Illumina short-read sequencing were performed in three biological replicates.

### Long-Read Sequencing

The library for Pacific Biosciences (PacBio; Menlo Park, CA, United States) Single Molecule Real Time (SMRT) sequencing was prepared from the aforementioned cDNAs. Cycle optimization was performed to determine the optimal number of cycles for the large PCRs. Three pools of cDNAs (1–2 kbp, 2–3 kbp, and 3–6 kbp) were prepared using the BluePipin size selection system. The SMRTbell library was configured using the SMRTbell Template Preparation Kit (PN 100-259-100). The DNA/Polymerase Binding Kit P6 (PacBio) was used for the DNA synthesis after the sequencing primers were annealed to the SMRTbell template. After the polymerase binding reaction, the library complex was combined with MagBeads and sequenced using the MagBead Kit. The MagBead-bound cDNA complex increases the number of reads per SMRT cell. This polymerization enzyme–SMRTbell–adapter complex was loaded into a zero-mode waveguide. The SMRTbell library was sequenced using eight SMRT cells (PacBio) with C4 chemistry (DNA Sequencing Reagent 4.0). Using the PacBio RS II sequencing platform, a 240-min movie was captured for each SMRT cell.

### *De novo* Transcriptome Assembly and Sequence Clustering

Raw transcriptomic data generated by the Illumina HiSeq were preprocessed to eliminate the nonsense sequences, including adapters, primers, and low-quality sequences (Phred quality score less than 20) using the NGS QC ToolKit (Patel and Jain, 2012). The raw data were further processed to remove ribosomal RNA using riboPicker v. 0.4.3 (Schmieder et al., 2012). The preprocessed reads were then assembled using Trinity (Haas et al., 2013), and the assembly statistics were calculated using an internal Perl script. The whole assembly was clustered to reduce the sequence redundancy (CD-HIT-EST v4.6.1) (Li and Godzik, 2006). Both the rank equality threshold and the alignment range (for short sequences) were set to 90% to create the clusters.

### Gene Family Identification

Genome and annotation data were downloaded for *Glycine max* (L.) Merr., *Medicago truncatula* Gaertn., *Mimosa pudica* L., and *Chamaecrista fasciculata* (Michx.) Greene. Gene models shorter than 10 amino acids or containing > 20% stop codons were removed, and OrthoMCL v. 2.0.3^1^ was used to perform a Markov clustering algorithm to define the cluster structures (Li et al., 2003). An all-versus-all analysis was conducted using BLASTP v. 2.2.25 + (*E*-value threshold 10^–5^). The protein pairs obtained from the BLASTP analysis were analyzed using the OrthoMCL program.

### Illumina Expression Quantification and Differential Expression Analysis

Clean reads from each tissue were aligned with an abundant transcriptome assembly using Bowtie2 (Langmead and Salzberg, 2012). The aligned reads were quantified as fragments per kilobase of transcript per million reads (FPKM) for the non-redundant combined transcript sequences (90% sequence similarity determined using CD-HIT-EST). Read coefficients for the alignment were performed using RSEM (RNA-Seq by Extraction Maximization) v. 1.2.25 (Li and Dewey, 2011). The differential expression analysis was performed using the DESeq2 package (Anders and Huber, 2010). Differentially expressed genes (DEGs) were identified between the tissues using a combination of a more than twofold change in expression and a significance (*P*-value) threshold of 0.001, based on the three biological replicates.

### Functional Annotation and Classification

All assembled unigenes were annotated through their comparison with the National Center for Biotechnology Information (NCBI) non-redundant (Nr) protein database, the Swiss-Prot protein database, and the Kyoto Encyclopedia of Genes and Genomes (KEGG) pathways database using the BLAST program (Altschul et al., 1990) with an *E*-value cutoff of 10^–5^. Whenever the results from the different databases conflicted, the results from the Swiss-Prot database were selected first, followed by the Nr database, and the KEGG database. A functional classification was performed using Gene Ontology (GO) terms (Ashburner et al., 2000) with the Blast2GO program (Conesa et al., 2005), with an *E*-value threshold of 10^–5^. AgriGO (Du et al., 2010) was used to identify any enrichment of the GO categories.

### Identification of Transcription Factor Families

To investigate the putative transcription factor (TF) families in *S. occidentalis*, the unigenes were mapped for all TF protein sequences available in the Plant Transcription Factor Database (PlantTFDB v. 4.0^2^) using BLASTX, with an *E*-value threshold of 10^–5^.

### qRT-PCR Analysis

Following the manufacturer’s instructions, an RNeasy Plant Mini Kit (Qiagen) was used to extract the total RNA. The quality of the extracted RNA was checked on an ethidium bromide–stained agarose gel, and the concentration was calculated according to the optical density (OD) of the specimen measured at 260 and 280 nm (DropSense96c Spectrophotometer; PerkinElmer, Waltham, MA, United States). Using the SuperScript III first strand RT-PCR kit (Thermo Fisher Scientific) with oligo(dT)_20_ primers, 1 μg of RNA was used for the cDNA synthesis. After obtaining cDNA from *S. occidentalis*, qRT-PCR was performed using gene-specific primers (Supplementary Table 1). The RT-PCR analysis was optimized and performed using a Roche LightCycler 480 II instrument (Roche, Basel, Switzerland) and SYBR Green Real-Time PCR Master Mix (Bio-Rad Laboratories, Hercules, CA, United States) under an initial denaturation of 95°C for 30 s, followed by 40 cycles of denaturation for 15 s and annealing and elongation at 55°C for 10 s. The relative expression of certain genes was quantified using the 2^–ΔΔ*Ct*^ calculation (Livak and Schmittgen, 2001), where ΔΔC_*t*_ is the difference in the threshold number of cycles. The internal reference gene for the data normalization was *ELONGATION FACTOR2*. The reliability of the amplification parameters was analyzed from a 1:15 dilution of the cDNA sample. The average threshold cycle value for the gene of interest was calculated from three experimental replications.

### Extraction of Anthraquinones and Liquid Chromatography–Mass Spectrometry Analysis

Young- and mature-stage seed samples were extracted with methanol using sonication for 30 min at 60°C. After extraction, the samples were centrifuged at 12,000 rpm for 3 min at 25°C, and the supernatant was filtered with 0.2-μm Acrodisc MS Syringe Filters with a WWPTFE membrane (Pall Corporation, Port Washington, NY, United States). A quantitative analysis of the anthraquinones was performed using a Triple TOF 5600 + spectrometer with a DuoSpray ion source (AB Sciex, Framingham, MA, United States) coupled with a Nexera X2 UHPLC (Shimadzu, Kyoto, Japan) equipped with a binary solvent manager, a sample manager, a column heater, and a photodiode array detector. UHPLC was performed on an ACQUITY UPLC BEH C18 column (1.7 μm, 2.1 × 100 mm, Waters Corporation, Milford, MA, United States). The mobile phases consisted of 5 mM ammonium acetate in water (eluent A) and 100% acetonitrile (eluent B). The gradient profile was as follows: 0–1 min, 20% eluent B; 1–3.5 min, 20–30% eluent B; 3.5–8 min, 30–50% eluent B; 8–12 min, 50–100% eluent B; and 11–17 min, 100% eluent B. The flow rate was 0.5 mL/min. A total of 5 μL of each sample was injected. To detect the peaks from the test samples, the following MS parameters in ESI-negative mode were used: nebulizing gas, 50 psi; heating gas, 50 psi; curtain gas, 25 psi; desolation temperature, 500°C; and ion spray voltage floating, 4.5 kV.

### Data Availability

The RNA-Seq and Iso-Seq sequences generated from the Illumina and PacBio RS II sequencing of the four tissue samples of *S. occidentalis* were deposited in the NCBI Sequence Read Archive database under the project number PRJNA631110.

## Results and Discussion

### RNA Sequencing and *de novo* Transcriptome Assembly

*De novo* transcriptome analysis is a great method for generating genetic information for an organism without performing additional genomic sequencing. It can lead to the discovery of new genes and molecular markers and can elucidate tissue-specific expression patterns. We sequenced cDNA libraries from the leaf, root, and seeds of *S. occidentalis* using the Illumina HiSeq 2500 system and the PacBio RS II platform. The Illumina sequencing platform produced 26,917,977,630 raw reads and averaged 2,243,164,802 reads per tissue (Supplementary Table 2). In total, more than 2 billion reads were of high quality (Q30-values over 81%). The Trinity assembler for the four different libraries generated 121,592 unigenes over 300 bp in length (Table 1). The length of the transcripts varied from 300 to 18,086 bp, with an average length of 827 bp, an N50 length of 918 bp, and a GC content of 39.0%.

**TABLE 1:** Assembly statistics of the *S. occidentalis* transcriptome by RNA-seq and Iso-Seq.

Assembly statistics	RNA-Seq	Iso-Seq
Number of unigenes	121,592	38,440
Total size (bp)	100,760,744	102,851,046
Minimum length (bp)	300	403
Maximum length (bp)	18,086	7,010
Average length (bp)	829	2,677
N50 length (bp)	918	3,232
GC contents (%)	39.00	38.60

The PacBio RS II sequencing platform produced 713,759 raw reads and 78,043 high-quality isoforms from three different libraries, which included 44,867, 34,858, and 42,274 high-quality isoforms for each of the sequencing sizes (< 2 kbp, 2–3 kbp, and > 3 kbp, respectively) (Supplementary Table 3). A total of 38,440 non-redundant unigenes were identified following the removal of redundant isoforms by the CD-HIT-EST program. The total size of the assembly was 102 Mbp, with a minimum unigene length of 403 bp and a maximum length of 7,010 bp. In total, our analysis generated two unigene sets: 121,592 unigenes from RNA-Seq and 38,440 unigenes from Iso-Seq (Table 1). Overall, the Iso-Seq produced longer reads than the RNA-Seq. The minimum length, average length, and N50 length were all confirmed to be longer for the Iso-Seq reads (Supplementary Figure 1); however, the GC contents for the assemblies produced by both sequencing methods were similar (Table 1).

### Functional Annotation and Classification

To characterize transcripts and understand the complexity and diversity of an organism, we need to annotate the function of the genes. For the functional annotation, the assembled 121,592 unigenes obtained using RNA-Seq from four samples (leaf, root, and young- and mature-stage seed tissues) were screened using an FPKM criterion of ≥ 1. To obtain the best annotation, the resulting 61,147 assembled RNA-Seq unigenes and 38,440 Iso-Seq unigenes from *S. occidentalis* were aligned with five public protein databases. We used the BLASTX program to search the unigene sequences against NCBI Nr, Clusters of Orthologous Groups of proteins (COGs), KEGG, GO, and Swiss-Prot databases with an *E*-value threshold of 1e–5, enabling the unigenes to be annotated with the function of matching known proteins. The Venn diagram in Supplementary Figure 2 displays the best BLASTX hits unique to the NCBI Nr, COG, KEGG, GO, and Swiss-Prot databases. A total of 43,809 of the RNA-Seq sequences were matched to the NCBI Nr database (66.28%), 53,965 to Swiss-Prot (81.64%), 50,952 to GO (77.08%), 24,171 to KEGG (36.57%), and 3,324 to COG (5.03%) (Supplementary Figure 2A), with the Swiss-Prot database providing the most matches. In contrast, 36,027 of the Iso-Seq sequences were matched to the NCBI Nr database (99.74%), 29,759 to Swiss-Prot (82.39%), 26,134 to GO (72.35%), 12,712 to KEGG (35.19%), and 6,473 to COG (17.92%) (Supplementary Figure 2B), with the most matches in the NCBI Nr database. New genes, long non-coding RNAs, or non-significant genes that could represent less-conserved untranslated regions were not evaluated in this annotation and require further analysis.

To further functionally characterize the *S. occidentalis* transcripts, we classified the functions of the RNA-Seq and Iso-Seq unigenes using a GO analysis. The distribution of the RNA-Seq and Iso-Seq unigene sets in different GO categories is shown in Supplementary Figure 3. The three main categories of GO annotation for RNA-Seq included 26,579 unigenes associated with GO terms for biological process (42.77%), 20,088 for molecular function (32.32%), and 15,482 for cellular component (24.91%). The most widely distributed GO terms in the main categories were 4,470 unigenes annotated with organic substance metabolic process functions in biological processes (17% of unigenes), with 3,739 and 3,736 unigenes associated with the GO terms of organic cyclic compound binding and heterocyclic compound binding, respectively, in molecular function (each 19% of unigenes), and 3,418 unigenes annotated as cell part and cells in cellular component (22% of unigenes). Conversely, for the Iso-Seq data, 58,931 unigenes were associated with GO terms for biological process (47.03%), 29,411 for molecular function (23.47%), and 36,973 for cellular component (29.50%). The most abundant GO categories in each main category were organic substance metabolic process in biological process (16% of unigenes), nucleotide binding and nucleoside phosphate binding in molecular function (16% of unigenes), and cell parts and cells in cellular component (24% of unigenes). This showed that the GO term patterns for RNA-Seq and Iso-Seq were similar (Supplementary Figure 3).

Members of TF families, such as ARF, bHLH, and C2H2, bind to specific regulatory elements in the promoters of their target genes to regulate their expression. TFs thus play a key regulatory role in the expression of genes involved in the biosynthesis and signaling of plant secondary metabolites and the response to environmental stress. The number of genes encoding different TFs varies between species as they are evolved to perform species-specific or developmentally specific functions (Yang et al., 2012). In this study, 6,422 unigenes (3,179 RNA-Seq and 3,243 Iso-Seq unigenes) were allocated to 58 TF groups. Among them, *bHLH* genes (544, 17.11%) were found to be the most abundant in the RNA-Seq dataset, followed by *WRKY*s (205, 6.45%), *MYB*s (185, 5.82%), and *bZIP*s (179, 5.63%). Similarly, *bHLH* genes (639, 19.70%) were also the most abundant TF group in the Iso-Seq dataset, although the next most abundant were *C3H*s (305, 9.40%), *bZIP*s (201, 6.20%), and *WRKY*s (193, 5.95%) (Supplementary Figure 4).

**FIGURE 1:**
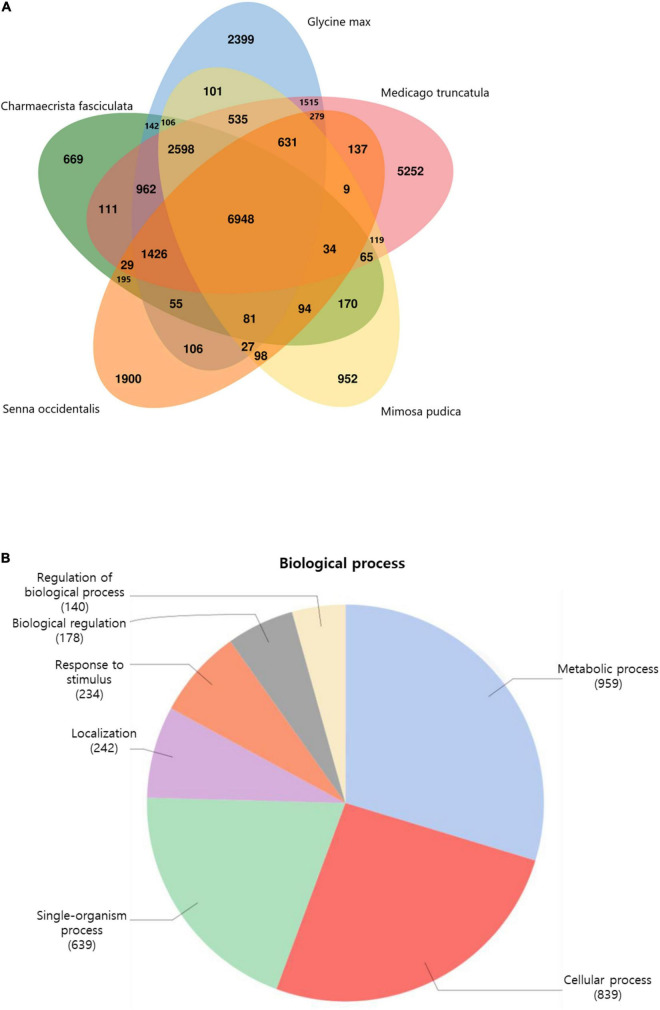
Histogram of gene family distribution between legume plants. **(A)** The gene family distribution between *Senna occidentalis* (Iso-Seq dataset) and other plants. **(B)** Gene Ontology analysis of the novel gene families of *S. occidentalis* associated with the biological process categories.

To study the relationships between *S. occidentalis* and other plant species, we categorized the transcripts into gene families and compared them with the gene families from four Fabaceae species: *Chamaecrista fasciculata*, *Mimosa pudica*, *Glycine max*, and *Medicago truncatula*. We found that 6,948 gene families were shared by all five species, whereas 1,900 gene families (containing 3,231 genes) were specific to *S. occidentalis* (Figure 1A). These *S. occidentalis*–specific genes included those encoding serine threonine-protein kinase, UDP-glucose 4-epimerase, transposons, and GATA TF family member proteins. A GO analysis of these novel gene families revealed that most of them are involved in metabolic processes and cell proliferation (Figure 1B). In addition, we showed that the *S. occidentalis* sequences showed a 38% similarity to those of *Glycine max*, 14% to *Phaseolus vulgaris*, and 9% to *Medicago truncatula*, suggesting that *S. occidentalis* was most closely related to *Glycine max* in the Fabaceae family (Supplementary Figure 5).

### Differential Gene Expression During Seed Development

The expression of genes varies depending on the species, developmental stage, or the environment to which the organism is exposed, and many genes are simultaneously expressed depending on the specific circumstances. Analyzing the expression levels of the genes encoding TFs is essential for understanding the transcriptional regulation of an organism. Accordingly, we studied tissue-specific TF gene expression in the leaves, roots, and developing seeds of *S. occidentalis* (Supplementary Table 4). Different TF expression patterns were observed in each *S. occidentalis* tissue. First, 68, 173, 99, and 50 TF-encoding genes were found to be expressed in the leaves, roots, and young- and mature-stage seeds, with 41, 102, 79, and 31 specific to each tissue, respectively. The TFs specifically expressed in the young seeds included members of the ARF, B3, CPP, LBD, Nin, and YABBY families, while in the mature-stage seeds, GRF and HD-ZIP family members were specifically expressed. Auxin response factors are TFs that play an important role in the expression of genes regulating the pathways for auxin, a plant hormone (Guilfoyle et al., 1998). In addition, the B3 TF family regulates various functions of plant growth and development (Peng and Weselake, 2013), while YABBY TFs are involved in a variety of activities, such as the response to plant hormone signals, stress tolerance, and the formation and development of reproductive organs (Zhang et al., 2020). On the contrary, GRF genes, which were specifically expressed in the mature seeds, are plant-specific TFs originally shown to function in stem and leaf development (Omidbakhshfard et al., 2015); however, recent research has emphasized their importance in other central developmental processes, including seed formation. The expression of *GRF* genes was also observed in rice (*Oryza sativa* L.) and maize (*Zea mays* L.), suggesting that it is involved in seed development (Zhang et al., 2008; Liu et al., 2012).

**FIGURE 2:**
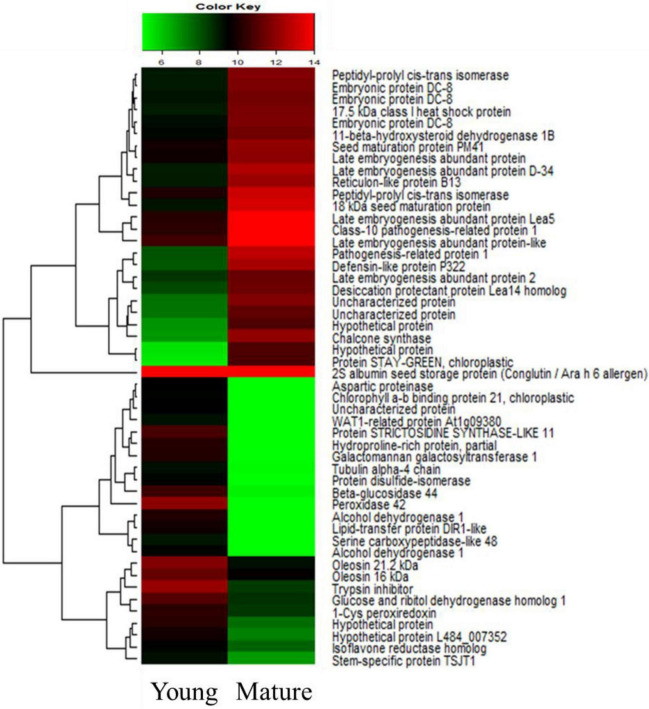
Heatmap of the top 25 up-regulated and 25 down-regulated genes according to the seed development stage of *Senna occidentalis.* The color bar indicates the value of the expression level in a sample.

To compare the differential expression of the *S. occidentalis* genes in mature-stage seed development compared with young-stage development, we used the DESeq method. The transcripts with a log_2_ fold change (FC) > 1 and a *p*-value < 1e–3 were considered to be DEGs. A pair-wise comparison of the transcripts from the young and mature stages of seed development resulted in 8,874 identified DEGs in the RNA-Seq dataset. As the seeds matured, 2,443 genes were upregulated and 6,431 genes were downregulated. These DEGs belong to diverse functional groups, including CHSs, dehydrogenases, carboxypeptidases, hormone-associated genes, and development-associated genes. A heatmap was constructed to cluster the top 25 up-regulated and 25 down-regulated genes based on the similarity and diversity of their expression profiles using normalized FPKM-values within a range of 6 to 16 (Figure 2). In the young stages of seed development, the expression of genes related to cellular components such as oleosin and tubulin alpha-4 are remarkably upregulated, and the expression of sugar-related genes such as galactomannan galactosyltransferase and beta-glucosidase were expressed at higher levels than in the mature stages. On the other hand, the genes involved in the development of seeds, such as those encoding embryonic proteins and late embryogenesis abundant proteins, were expressed at a higher level in the mature stage of seed development. In addition, it was confirmed that *CHS* was expressed at a high level in the mature-stage seed, likely to create substances for the protection of the seeds.

**FIGURE 3:**
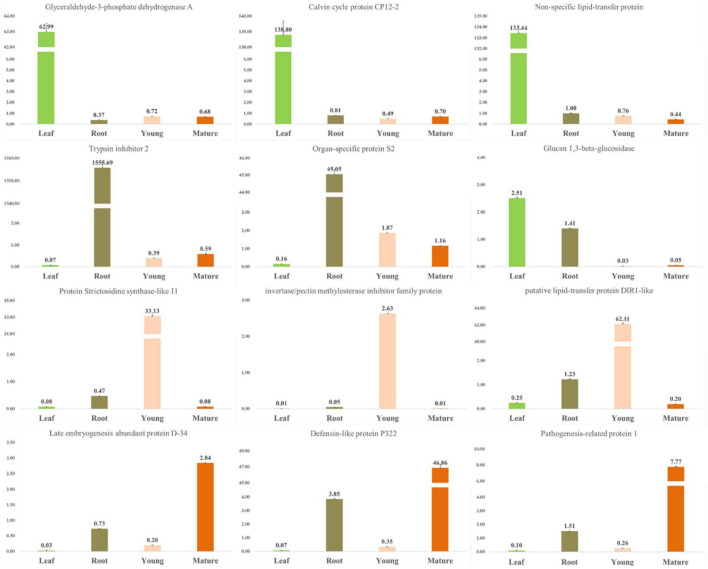
qRT-PCR validation of tissue-specific unigene expression levels. All RT-PCR experiments were performed at least three times in each independent biological experiment (three replicates). Error bars represent the SEM.

Similarly, the tissue-specific gene expression was determined in the leaves, roots, and young- and mature-seeds tissues. Transcripts exhibiting tissue-specific expression were identified and the top 10 transcripts were selected (Supplementary Figure 6). A qRT-PCR analysis was performed to accurately identify the differential expression of selected transcripts belonging to the carbohydrate metabolic pathway, the secondary metabolite pathway, and the associated TFs (Figure 3). The tissue-specific gene expression data were congruous in various tissues. Glyceraldehyde 3-phosphate dehydrogenase has a catalytic role in glycolysis and plays a crucial role in plant metabolism and development (Muñoz-Bertomeu et al., 2010). The glucan 1,3-beta-glucosidase gene exhibited the highest expression level in the leaf, followed by the root, mature seed, and young seed. The gene encoding the late embryogenesis abundant protein showed the highest expression level in the mature seed, followed by the root, young seed, and leaves. The genes encoding the defensin-like protein and pathogenesis-related-like protein were most highly expressed in the mature seed, followed by the root, young seed, and leaves. The gene encoding strictosidine synthase-like protein 11 was most highly expressed in the young seed, followed by the root. Strictosidine synthase is an enzyme that biosynthesizes strictosidine through the Pictet–Spengler reaction using tryptamine and secologanin as substrates and is important in alkaloid biosynthesis (Maresh et al., 2008).

To determine the biological function of the DEGs in seed development, a GO classification analysis was performed using the Blast2GO program. A total of 62,149 unigenes were categorized into 25 functional groups, including three major ontologies, with annotations added based on the GO database. In terms of the biological process terms, most of the DEGs were classified into the organic substance metabolic process function; for molecular functions, organic cyclic compound binding was the most common term, while cell parts and cells dominated the cellular components category. The orthologous *S. occidentalis* genes were subjected to a GO enrichment analysis using the AgriGO program to determine whether the transcripts were enriched in functions essential for seed development. The carbohydrate metabolic process GO term was highly enriched in the DEGs downregulated in the seeds (Supplementary Figure 7). In addition, the lipid metabolic process GO term (biological process) was enriched in the young seed (Supplementary Figure 8).

**FIGURE 4:**
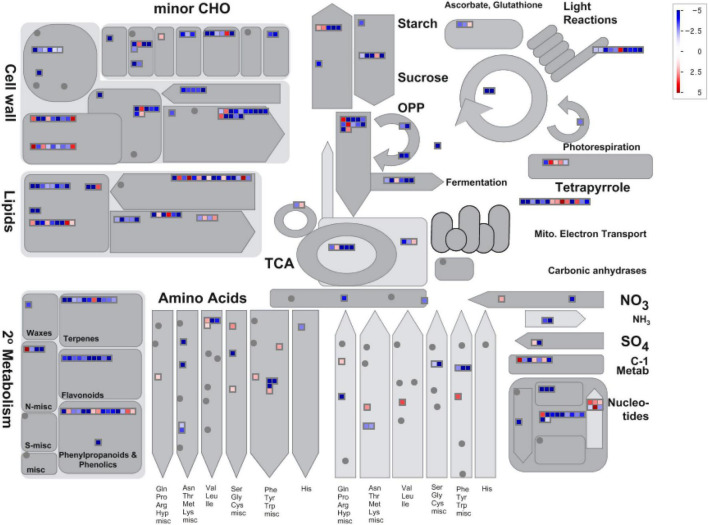
MapMan metabolism overview maps showing differences in transcript levels between young- and mature-stage seeds. MapMan software was used to provide a snapshot of the modulated genes in the main metabolic pathways. Log_2_ fold-change values are represented. Upregulated and downregulated transcripts are shown in red and blue, respectively.

To identify the specific metabolic pathway responsible for the changes in gene expression during the process of seed development in *S. occidentalis*, a MapMan (Thimm et al., 2004) analysis was performed using the expression data of genes showing at least a twofold difference in expression between the young and mature seeds. We designed a diagram of the biological process required for the analysis, and based on this, the log_2_ normalized expression value was displayed in the diagram (Figure 4). The results of the MapMan v. 3.5.1R2 mapping of the annotated genes revealed an enrichment in functions associated with the cell wall, lipids, and secondary metabolism, such as phenylpropanoids, terpenes, and flavonoids. In terms of the cell wall, genes related to pectin-esterase and UDP-sugar metabolism were all shown to be downstream, and in terms of cell wall breakdown, genes related to the pectin-lyases and polygalacturonase were downstream except for two genes. In the case of lipids, the results were varied. All five genes annotated as GDSL lipase were downstream, whereas some beta-oxidation and phospholipase genes were upstream. In addition, the fatty acid synthase genes largely functioned downstream, but six genes were shown to be upstream. Genes involved in the secondary metabolism were largely downstream, while some genes associated with phenylpropanoids, phenolics, and terpenes functioned upstream.

### Candidate Gene Families Involved in Anthraquinone Biosynthesis

*Senna occidentalis* is used in traditional medicine in many countries around the world. Each of its medicinal uses (e.g., as a laxative, analgesic, expectorant, diuretic, etc.) derives from its various secondary metabolites, the best known of which are anthraquinones, alkaloids, flavonoids, and terpenoids. In addition, toxicological studies have shown that *S. occidentalis* has myotoxic, hepatotoxic, and neurotoxic effects that can harm laboratory animals (Gotardo et al., 2017). The major toxic component of this plant’s seeds is dianthrone, a compound derived from anthraquinone (Haraguchi et al., 1998, 2003). Anthraquinone derivatives are biosynthesized through the isochorismate pathway, which is shared with the biosynthesis pathways of the phenylpropanoids and the MEP/DOXP, MEV, as well as the shikimate biosynthesis pathways, shared with the carotenoid and flavonoid biosynthesis pathways. In addition, the polyketide pathway, involving PKSs such as CHS, is an important part of anthraquinone biosynthesis (Kang et al., 2020b).

To analyze anthraquinone biosynthesis, we determined the levels of seven derivatives of the anthraquinone biosynthesis pathway in the young- and mature-stage seeds and compared them with those identified in *S. tora* in previous studies (Table 2; Kang et al., 2020a). Three of the derivatives (obtusin, chryso-obtusin, and obtusifolin) isolated from *S. tora* could not be isolated from *S. occidentalis* or were present only in trace amounts. Citreorosein was isolated from *S. occidentalis* at a concentration of 6.91 ± 2.65 μg/g in the mature-stage seeds. Physcion was isolated from both *S. tora* and *S. occidentalis*, but was more abundant in the latter. The differences in the concentrations of each of the seven derivatives between the two species is caused by the differences in their compositions of the genes involved in the anthraquinone biosynthesis pathway. The total amount tended to decrease over time, as more was isolated from the young seeds than the mature seeds; however, aurantio-obtusin maintained a constant concentration in *S. tora* regardless of the stage of seed development, while citreorosein accumulated in higher quantities in the mature-stage *S. occidentalis* seeds.

**TABLE 2:** Summary of the anthraquinone derivative concentration between *S. occidentalis* and *S. tora*.

Structure	Name	Concentration Mean (μg/g)			
		*Senna occidentalis*		Senna tora*	
		Young	Mature	Young	Mature
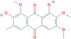	Obtusin	N/D	0.01	0.83 ° 0.00	0.60 ° 0.04
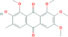	Chryso-obtusin	N/D	N/D	1.39 ° 0.40	0.69 ° 0.11
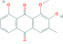	Obtusifolin	N/D	N/D	1.38 ° 0.27	0.26 ° 0.06
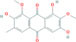	Aurantio-obtusin	0.39 ° 0.31	0.27 ° 0.18	1.07 ° 0.38	1.08 ° 0.13
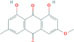	Physcion	14.72 ° 2.22	7.86 ° 3.02	2.02 ° 0.84	0.960.65
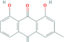	0.38 ° 0.35	0.23 ° 0.14	11.39 ° 4.22	6.69 ° 4.86
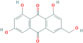	Citreorosein	1.04 ° 0.10	6.91 ° 2.65	N/A	N/A

*Reference: previous quantitative analysis of anthraquinone in Senna tora (Kang et al., 2020a). N/D, Not detected; N/A, Not assayed.

**FIGURE 5:**
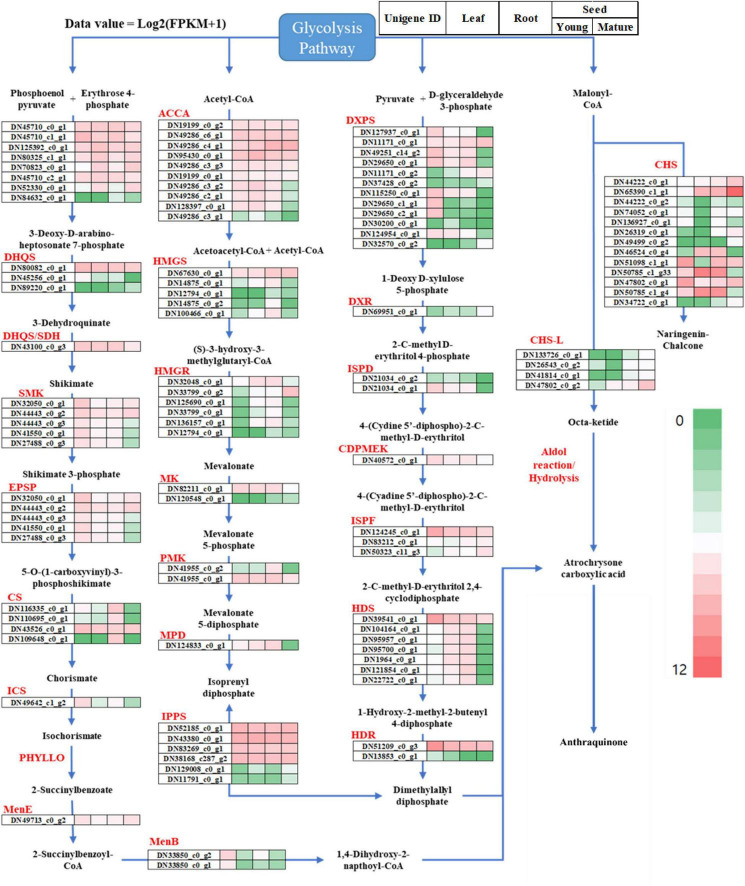
Overview of the putative anthraquinone biosynthesis pathway genes in *Senna occidentalis.* The gene expression levels were normalized to the fragments per kilobase of transcript per million reads (FPKM) values to compare the changes in expression between different tissues of *S. occidentalis*. The total gene expression levels were clustered based on log_2_ (FPKM + 1).

To observe the differential gene expression levels in the leaves, roots, and seeds, the expression levels were normalized to the FPKM-values, and the transcripts were hierarchically clustered based on the log_2_ (FPKM + 1) value (Figure 5). In our study, 582 RNA-Seq unigenes were involved in the *S. occidentalis* secondary metabolite pathways, which were classified into five groups (the MEP/DOXP, MEV, shikimate, carotenoid, and flavonoid/polyketide pathways) based on the anthraquinone biosynthesis pathway of *S. tora* (Figure 5 and Supplementary Table 5; Kang et al., 2020a). For the MEP/DOXP pathway, which led to the production of the precursor dimethylallyl diphosphate, 28 and 32 unigenes from the RNA-Seq and Iso-Seq datasets, respectively, were identified. In addition, there were 27 and 30 unigenes for RNA-Seq and Iso-Seq, respectively, in the MEV pathway, which produces the same precursor as the MEP/DOXP pathway. For genes belonging to both pathways, more than one unigene could be identified in each tissue and seed development stage, and all the unigenes (except those with consistently high expression levels) showed low expression levels in the mature-stage seeds. Equally, 27 and 30 unigenes belonging to the shikimate pathway were identified in the RNA-Seq and Iso-Seq datasets, respectively. Multiple Iso-Seq unigenes were identified for some genes, whereas in the RNA-Seq dataset, PHYLLO (2-succinyl-5-enolpyruvyl-6-hydroxy-3-cyclohexene-1-carboxylate synthase) was not detected. Again, the expression level of each gene showed a tendency to be downregulated in the mature-stage seeds, except those genes with high expression levels in all tissues and throughout seed development. The precursors of the *S. occidentalis* secondary metabolites are believed to be generated in the young stages of seed development, and these precursors downregulate gene expression as the seed moves into the late developmental stages.

Based on previous studies, many researchers predicted that anthraquinone might be biosynthesized in plants through the polyketide biosynthesis pathway, the most important enzyme of which is CHS, a type III PKS (Pandith et al., 2016). It was also suggested that PKS could form an anthraquinone precursor, octaketide, using acetyl-CoA and malonyl-CoA, which would then be cyclized by PKC-encoding polyketide cyclase to form three-ring structures with A, B, and C rings (Rama Reddy et al., 2015). In fact, CHS-L was recently found to catalyze the production of atrochrysone carboxylic acid, a precursor of anthraquinones found in *S. tora* (Kang et al., 2020b). Of the 16 CHS-L proteins identified in that species, Sto07g228250 (CHS-L9) was found to biosynthesize atrochrysone carboxylic acid and endocrocin anthrone using malonyl-CoA as substrates. Based on this, we identified 28 RNA-Seq unigenes encoding type III PKSs such as CHS and STS (Supplementary Table 5). These unigenes averaged 609 bp in length, with the shortest being 301 bp and the longest being 1,715 bp. To identify the *CHS-L* unigene involved in the biosynthesis of anthraquinone, we created a phylogenetic tree to compare these sequences against the *CHS-L* gene of *S. tora* (Sto07g228250) and the *CHS2* gene of *Medicago sativa* (L.). To enhance the accuracy of the analysis, only the 17 genes that encode proteins longer than 100 amino acids were used; the remaining 11 genes were excluded from the analysis (Figure 5). DN133726_c0_g1, DN26543_c0_g1, DN41814_c0_g1, and DN47802_c0_g2 were found to share sequence similarity with *CHS-L* (Sto07g228250), with the remaining 13 genes being classified as *CHS*. Among the *CHS-L* orthologs, DN47802_c0_g2 was more highly expressed in the mature stage than in the young stage of seed development, with the other genes showing a similar pattern or an even higher expression difference. In addition, DN47802_c0_g2 was not expressed in the leaves or roots, or was only present in very small amounts. In the case of the *CHS* genes, DN65390_C1_g1 showed very high expression levels in the mature-stage seed, and DN50785_c1_g33 and DN50785_c1_g4 showed the highest expression in the root.

**FIGURE 6:**
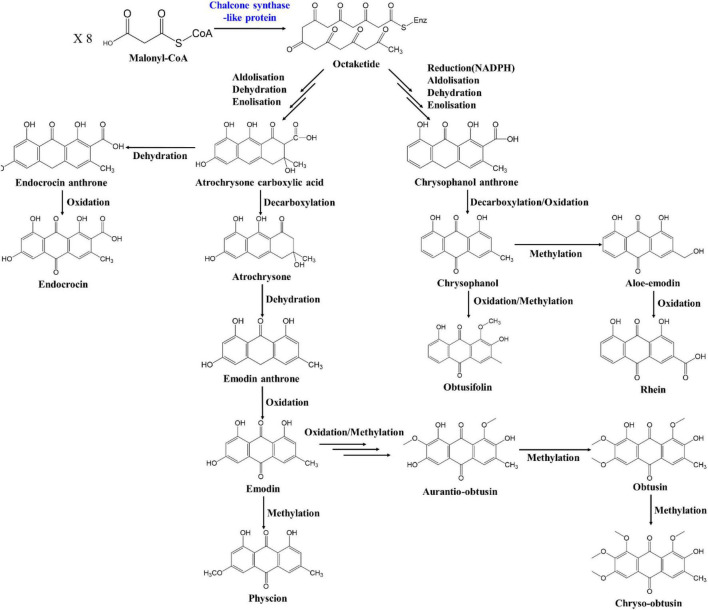
Putative anthraquinone biosynthesis pathways in *Senna occidentalis.*

The anthraquinone biosynthesis pathway exists as two major pathways, the chorismite/*O*-succinyl benzoic acid pathway and the polyketide pathway, as described above. However, the rest of this pathway has not been elucidated in plants such as *Senna* and rhubarb (*Rheum* sp. L.) after the backbone of anthraquinone has been formed. The full anthraquinone pathway is not yet understood, except in bacteria and fungi, which produce anthranoid or xanthone compounds (Zhou et al., 2019). Many of the genes in bacteria and fungi may have similarities with those in plants, however. In addition, as the flavonoid biosynthesis pathway in plants shares the polyketide pathway, several similarities may exist between the anthraquinone and flavonoid biosynthesis pathways (Liu et al., 2020). Based on the principal component of *S. occidentalis* and previous studies of the anthraquinone biosynthesis pathway, we propose a putative anthraquinone biosynthesis pathway including the anthraquinone derivatives, as shown in Figure 6. The polyketide backbone formation, oxidation, methylation, and glycosylation that occur in the biosynthesis of anthraquinone derivatives exist in all the above processes, suggesting that the biosynthesis of compounds can share the same functional enzymes.

### Candidate Gene Families Involved in the Modification of Anthraquinone Derivatives

Plants produce secondary metabolites by first producing the backbone molecular structure and then converting this backbone into a functional substance using specific structure-modifying enzymes. The modifying enzymes involved in this pathway include cytochrome P450 (CYP) and glycosyltransferase (GT). Among these, the CYP superfamily is a group of heme proteins that play important roles in promoting plant growth, development, and protection from stress through several biosynthesis and detoxification pathways (Ohkawa et al., 1998; Li et al., 2012). Here, we identified 180 CYPs (Supplementary Table 5) in the RNA-Seq of *S. occidentalis* and 50 CYPs in the Iso-Seq dataset. The overall average length of the putative *CYP*s was 1,213 bp, the longest was 3,076 bp, and the shortest was 301 bp. Our analysis of these unigenes based on their annotation using NCBI Nr revealed that the most commonly identified CYP groups were CYP71, CYP94, CYP82, and CYP89, with 39, 18, 14, and 10 unigene representatives, respectively. In addition, six cytochrome P450 reductases, an important component of the catalytic activity of CYP, were also identified. Among these unigenes, some of the CYP unigenes were specifically expressed at distinct stages of seed development. DN50163_c9_g1, DN50163_c8_g3, and DN95508_c0_g1 appeared to be expressed in both young and mature seeds, but showed higher expression levels in the mature-stage seeds. Conversely, DN125836_c0_g1, DN17292_c0_g1, DN50624_c1_g1, DN95253_c0_g1, and DN41954_c0_g2 showed higher expression levels in the young-stage seeds than the mature-stage seeds.

Glycosylation is widespread in nature and is involved in almost all important processes. In general, it refers to saccharide polymerization or the binding of sugar molecules to other biomolecules, including proteins, lipids, nucleic acids, and small natural molecules. These glycosylated compounds possess a wide range of functions, including energy storage, the maintenance of cellular structural integrity, information storage and transfer, molecular recognition, the regulation of cell–cell interactions, viral immune responses, and chemical defense (Liang et al., 2015). Glycosylation occurs at the end of secondary metabolite biosynthesis and improves the solubility and stability of the compounds. These glycosylated compounds are structurally the most diverse biomolecules, and their biosynthesis requires complex biological processes regulated by many enzymatic systems. This glycosylation is catalyzed by GTs, which mediate the formation of stereo-specific glycosidic bonds and regions between the sugar moieties and various important biomolecules. In nature, UDP-glycosyltransferases (UGTs) normally facilitate glycosylation, generating a product with a glucose moiety at the hydroxyl group (Ross et al., 2001). Here, we identified 157 putative *GT* genes in the *S. occidentalis* transcripts. In addition, nine, three, four, and two unigenes were specifically expressed in the leaves, roots, young-stage seeds, and mature-stage seeds, respectively. In particular, DN80158_c0_g1 showed the highest expression level among the tissue-specific *GT*s, while DN48605_c0_g1 was the most highly expressed unigene in the mature-stage seeds. Specifically, this unigene showed a rapid change in expression level from 8.32 in the young-stage seeds to 208.66 in the mature-stage seeds. After the anthraquinone derivative is formed, glycosylation is required for them to accumulate them in the seed, which suggests that this gene may be vital for such a role.

### Comparison of Candidate Genes for Modifying the Anthraquinone Skeleton in *Senna occidentalis* and *Senna tora*

The biosynthesis of the anthraquinone backbone is similar in *S. occidentalis* and *S. tora*, although more unigenes related to this function were identified in *S. tora* than in *S. occidentalis* (Supplementary Table 6). In addition, the PHYLLO unigene, 2-succinyl-5-enolpyruvyl-6-hydroxy-3-cyclohexene-1-carboxylate synthase in the shikimate pathway, could not be identified in RNA-Seq dataset in either species, but two and four PHYLLO unigenes were found in the Iso-Seq datasets for *S. occidentalis* and *S. tora*, respectively. Similar patterns were observed in the expression levels in the RNA-Seq data from the two species; however, isopentenyl diphosphate delta-isomerase, which is responsible for the conversion of isoprenyl diphosphate and dimethylallyl diphosphate in the MEV and MEP/DOXP pathways, was more highly expressed in *S. occidentalis* than *S. tora*. In addition, the expression of chorismite synthase in the shikimate pathway was equally high in the young-stage seeds of *S. occidentalis* and *S. tora*, but *S. occidentalis* showed higher levels in the mature-stage seeds.

In the RNA-Seq data for *S. occidentalis*, 28 type III PKSs were identified, whereas only 27 were found in *S. tora*. On the other hand, the Iso-Seq data showed 23 type III PKSs in *S. tora* and 22 in *S. occidentalis.* Some of the type III PKSs in *S. tora* were classified as CHS-Ls, similar to CHSs, which form the backbone of anthraquinone compounds called atrochrysone carboxylic acid. Following our classification of the type III PKSs in *S. occidentalis* based on the *S. tora* genome (Kang et al., 2020b), only 4 of the 28 genes were classified as *CHS-L*s in *S. occidentalis*, while in *S. tora* 16 genes were considered *CHS-L*s. The *CHS*s in *S. tora* were more highly expressed in the young stages of seed development, whereas in *S. occidentalis* they showed high expression in the young and mature stages. This suggests that the types and amounts of anthraquinone derivatives that accumulate in *S. occidentalis* will be different from those in *S. tora*. In the RNA-Seq dataset, the expression patterns of *S. occidentalis* and *S. tora* suggest that the polyketide pathway was preferred over the chorismite/*O*-succinylbenzoyl acid pathway due to the absence of PHYLLO and the inconsistency of the expression patterns and product patterns of the pathway-associated unigenes. In addition, *CYP* and *UGT* were differently expressed in *S. occidentalis* and *S. tora*. A total of 180 and 194 genes were identified in the RNA-Seq datasets of *S. occidentalis* and *S. tora*, respectively, whereas 65 and 71 genes were found in the Iso-Seq dataset. There were no significant differences in the *CYP* expression patterns between species, but a significant difference was observed in the *UGT* genes. The modifying enzyme genes involved in anthraquinone biosynthesis have not yet been identified yet.

This study suggested that CHS-L proteins are responsible for producing anthraquinones in *S. occidentalis*, as a similar function was determined for orthologous genes in *S. tora* in our previous study (Kang et al., 2020b). Variation in the anthraquinone production between different developmental stages, tissues, and species, including *S. occidentalis* and *S. tora*, should be further explored. In addition, the expression of this metabolite’s structure-modifying enzymes, CYP and UGT, was demonstrated in *S. occidentalis* and *S. tora*, providing an insight into secondary metabolite formation and the resistance to stress in plants. Hence, our transcript analysis for *S. occidentalis* and *S. tora* could lead to the identification of various secondary metabolite biosynthesis pathways and their regulatory mechanisms.

## Data Availability Statement

The datasets presented in this study can be found in online repositories. The names of the repository/repositories and accession number(s) can be found in the article/Supplementary Material.

## Author Contributions

S-HK conceived of and supervised the project. S-HK and J-SS contributed to the sample preparation, sequencing, and metabolite analysis. S-HK and W-HL performed the transcriptome data analysis. NT, SC, and J-PH participated in the methodology. S-HK and W-HL wrote the manuscript. S-HK and T-JO revised the manuscript. All authors read and approved the manuscript.

## Conflict of Interest

The authors declare that the research was conducted in the absence of any commercial or financial relationships that could be construed as a potential conflict of interest.

## Publisher’s Note

All claims expressed in this article are solely those of the authors and do not necessarily represent those of their affiliated organizations, or those of the publisher, the editors and the reviewers. Any product that may be evaluated in this article, or claim that may be made by its manufacturer, is not guaranteed or endorsed by the publisher.

## Footnotes

1
http://orthomcl.org/orthomcl/

2
http://planttfdb.gao-lab.org/download.php

## Supplementary Material

Supplementary Figure 1 The length distribution of transcripts in *S. occidentalis.*

Supplementary Figure 2 The distribution of annotated unigenes using various public protein databases. Venn diagrams display the proportion of unigenes annotated using the NCBI Nr, KEGG, Swiss-Prot, and GO databases.

Supplementary Figure 3 Histogram of Gene Ontology classifications. The results are summarized in the three main categories: biological process, molecular function, and cellular component.

Supplementary Figure 4 Distribution of the transcription factor (TF) families in *S. occidentalis.* Distribution of transcripts (3,179 for RNA-Seq and 3,243 for Iso-Seq) that encode for TFs.

Supplementary Figure 5 Species distribution of the top BLAST hits. The most frequently hit species using the RNA-Seq and Iso-Seq datasets were calculated based on sequence alignments with the lowest *E*-value obtained from BLAST.

Supplementary Figure 6 Heatmaps representing the top 10 genes with tissue-specific expression in the *S. occidentalis* leaf, root, and young- and mature-stage seeds. Red and green represent high and low abundance, respectively.

Supplementary Figure 7 AgriGO analysis of the downregulated genes during seed development. The downregulated genes associated with molecular terms are represented by increasingly red colors. A Gene Ontology (GO) term enrichment analysis was performed using the single enrichment analysis (SEA) tool in AgriGO. Box colors indicate the levels of statistical significance: yellow = 0.05; orange = e–05; and red = e–09.

Supplementary Figure 8 AgriGO analysis of tissue-specific genes during seed development. Tissue-specific genes (A, leaf; B, root; and C, young_seed) associated with molecular terms are represented by increasingly red colors. A Gene Ontology (GO) term enrichment analysis was performed using the single enrichment analysis (SEA) tool in AgriGO. Box colors indicates the levels of statistical significance: yellow = 0.05; orange = e–05; and red = e–09.

Supplementary Table 1 Gene-specific primers used for tissue-specific qRT-PCR.

Supplementary Table 2 General properties of the reads produced using the Illumina HiSeq 2500 sequencing platform.

Supplementary Table 3 General properties of the reads produced using the PacBio sequencing platform.

Supplementary Table 4 Distribution of the tissue-enriched and tissue-specific transcription factors in each tissue.

Supplementary Table 5 Genes associated with the secondary metabolite pathway in *S. occidentalis.*

Supplementary Table 6 Comparison of the number of genes involved in the anthraquinone biosynthesis pathway in *S. tora* and *S. occidentalis*.
