# Supplementary figures and images for: De novo Transcriptome Assembly of Senna occidentalis Sheds Light on the Anthraquinone Biosynthesis Pathway
Source: Front Plant Sci. 2022 Jan 3;12:773553. doi: 10.3389/fpls.2021.773553 (PMC8761625; doi:10.3389/fpls.2021.773553)

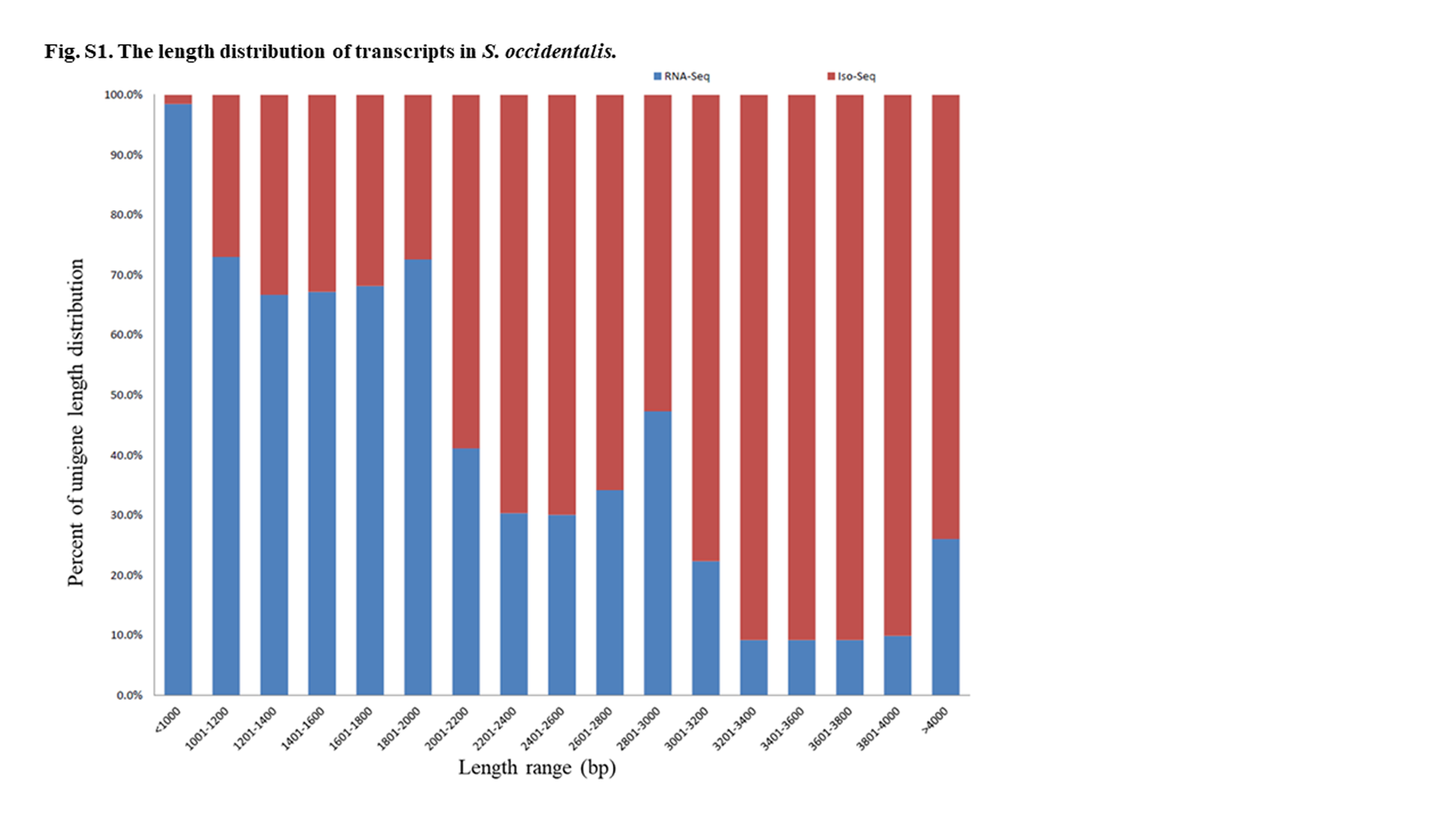

Supplement: Supplementary Figure 1 — The length distribution of transcripts in S. occidentalis. [file Image_1.tif]

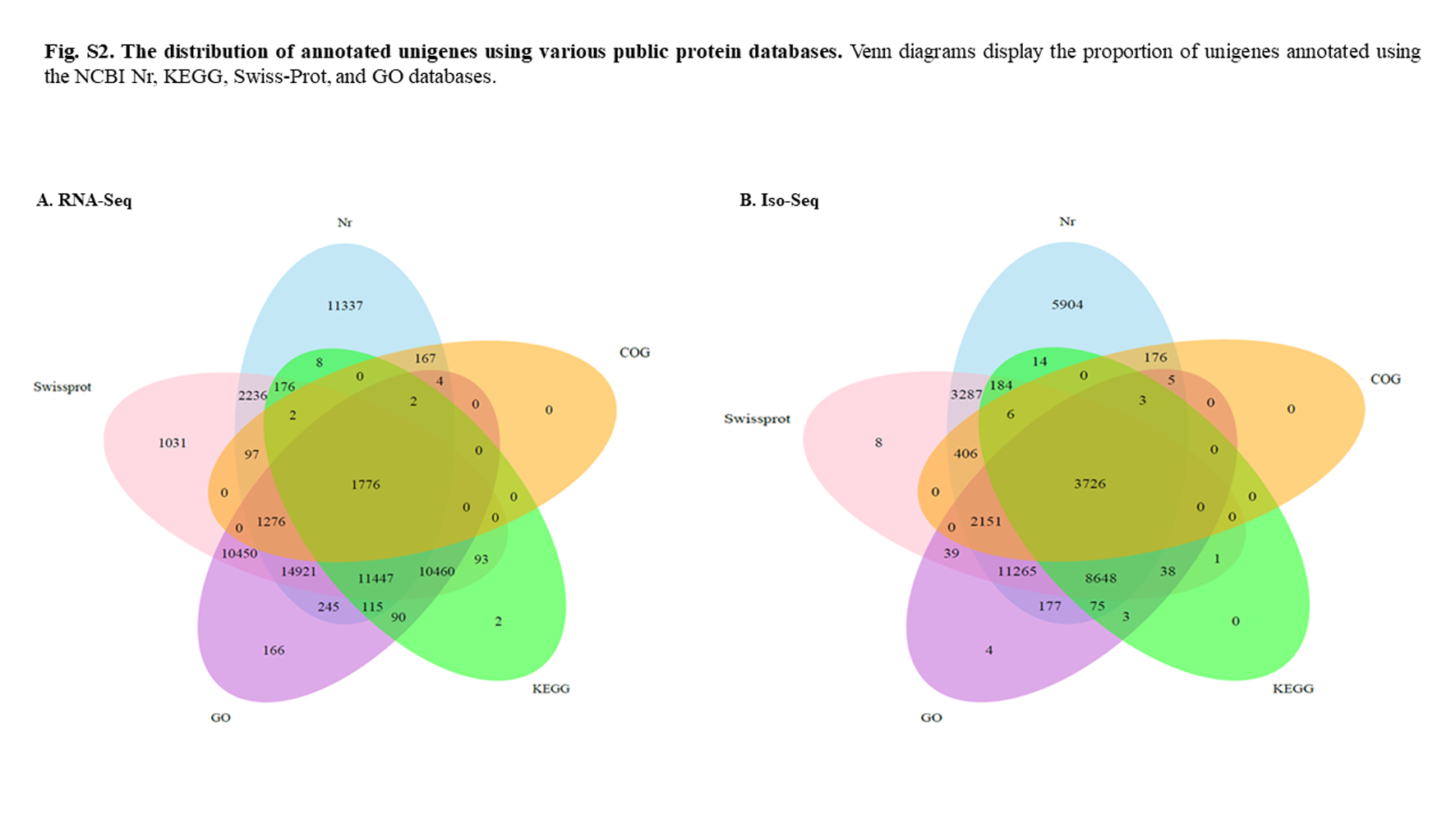

Supplement: Supplementary Figure 2 — The distribution of annotated unigenes using various public protein databases. Venn diagrams display the proportion of unigenes annotated using the NCBI Nr, KEGG, Swiss-Prot, and GO databases. [file Image_2.tif]

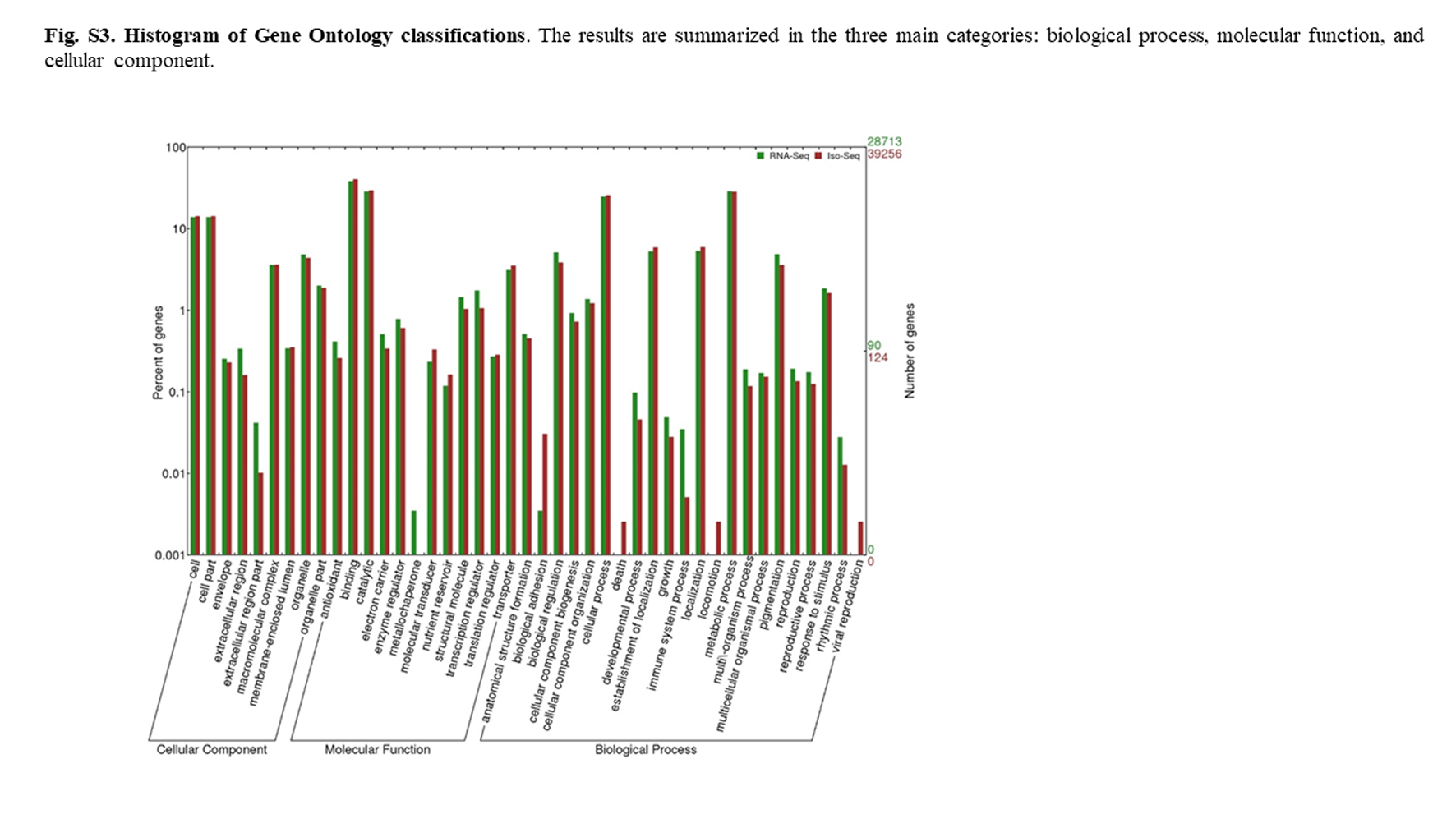

Supplement: Supplementary Figure 3 — Histogram of Gene Ontology classifications. The results are summarized in the three main categories: biological process, molecular function, and cellular component. [file Image_3.tif]

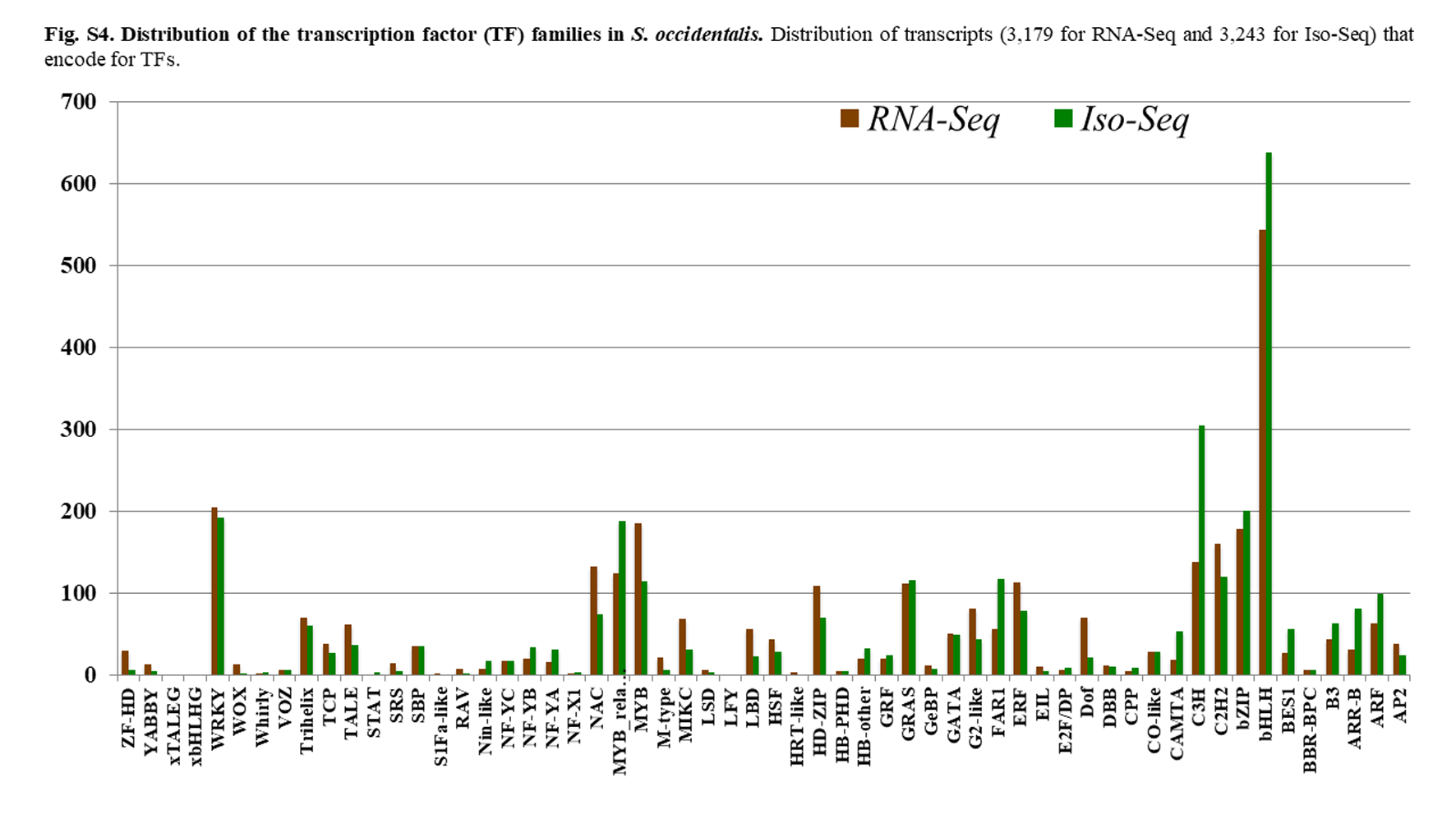

Supplement: Supplementary Figure 4 — Distribution of the transcription factor (TF) families in S. occidentalis. Distribution of transcripts (3,179 for RNA-Seq and 3,243 for Iso-Seq) that encode for TFs. [file Image_4.tif]

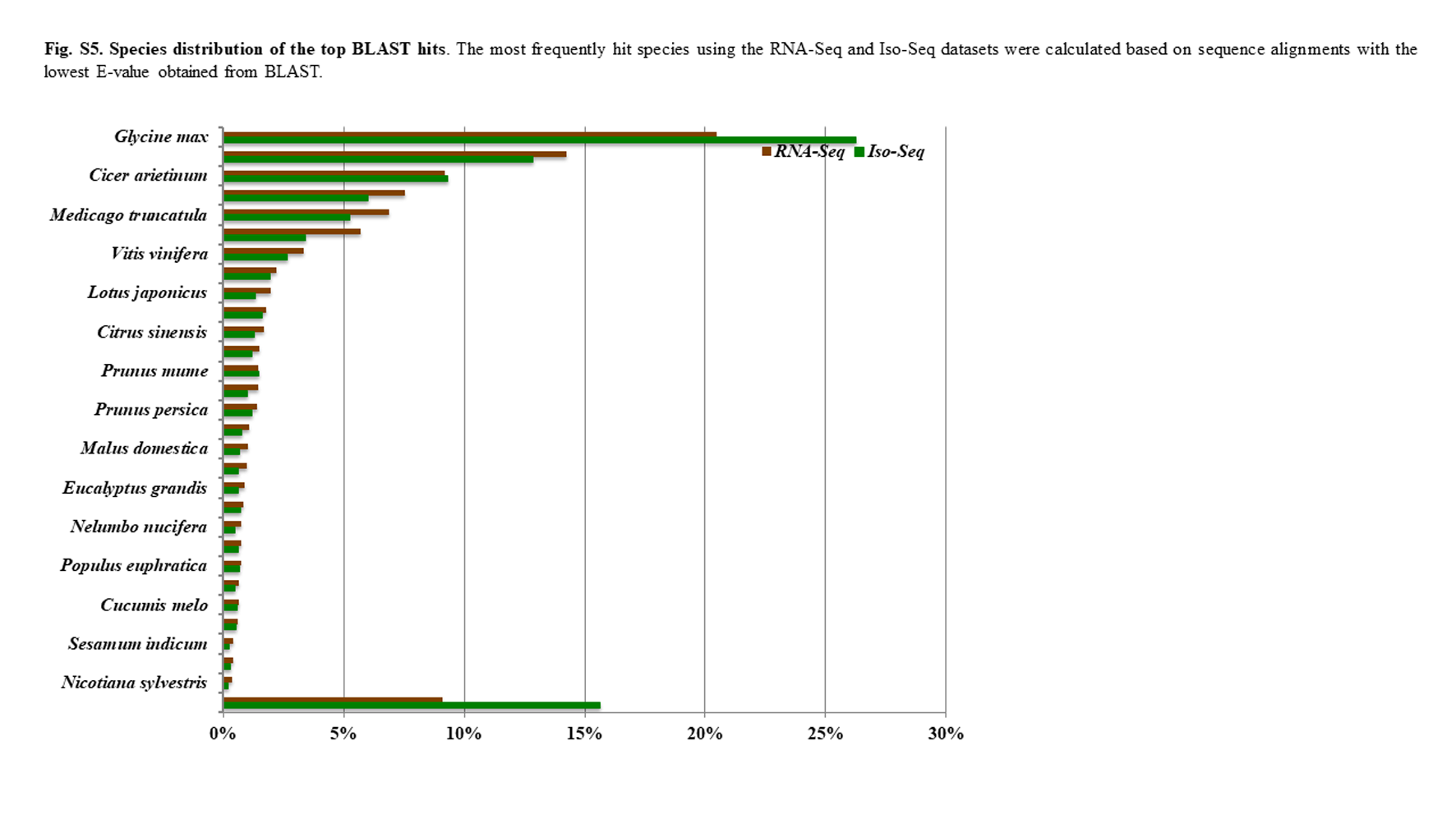

Supplement: Supplementary Figure 5 — Species distribution of the top BLAST hits. The most frequently hit species using the RNA-Seq and Iso-Seq datasets were calculated based on sequence alignments with the lowest E-value obtained from BLAST. [file Image_5.tif]

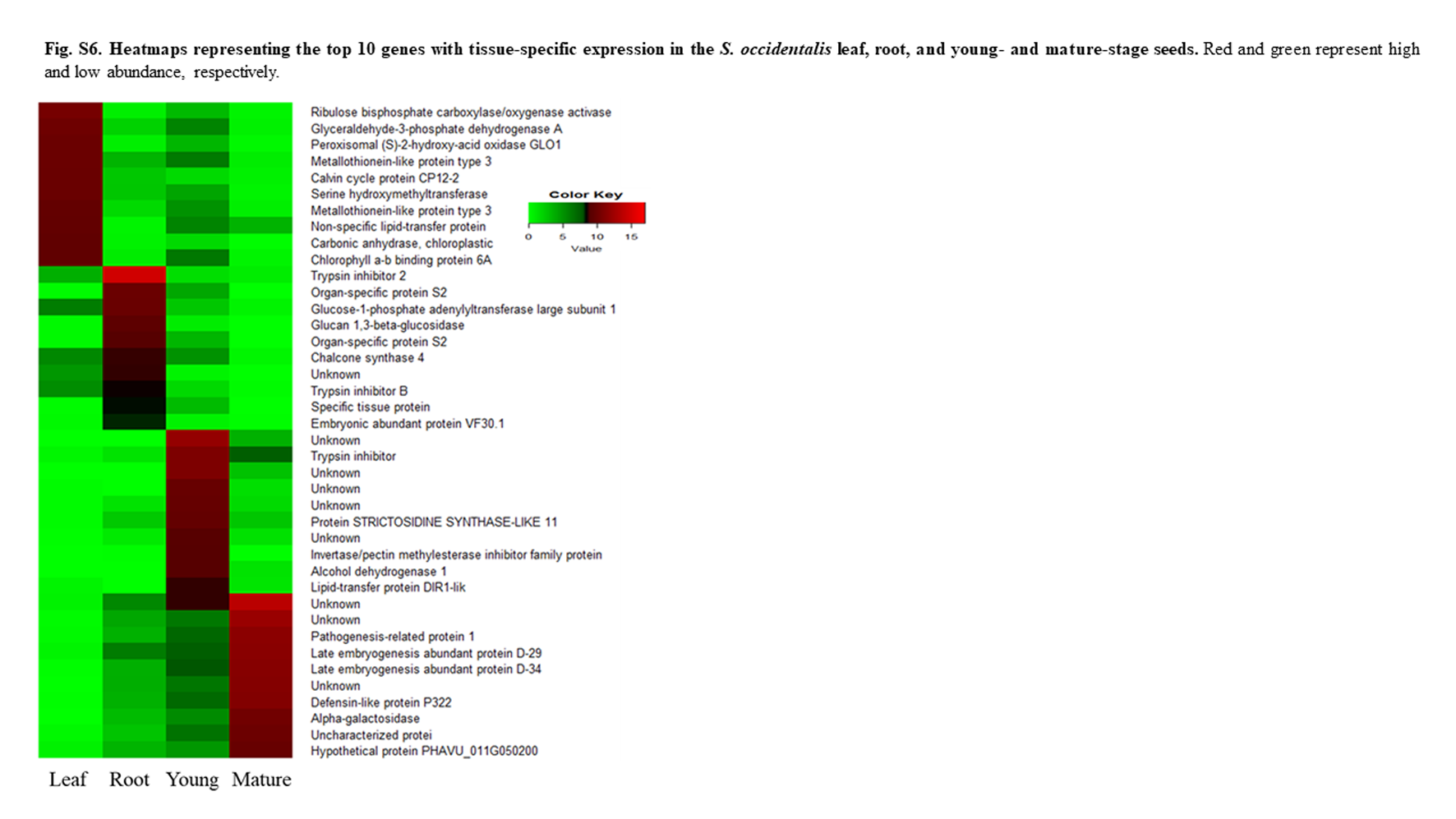

Supplement: Supplementary Figure 6 — Heatmaps representing the top 10 genes with tissue-specific expression in the S. occidentalis leaf, root, and young- and mature-stage seeds. Red and green represent high and low abundance, respectively. [file Image_6.tif]

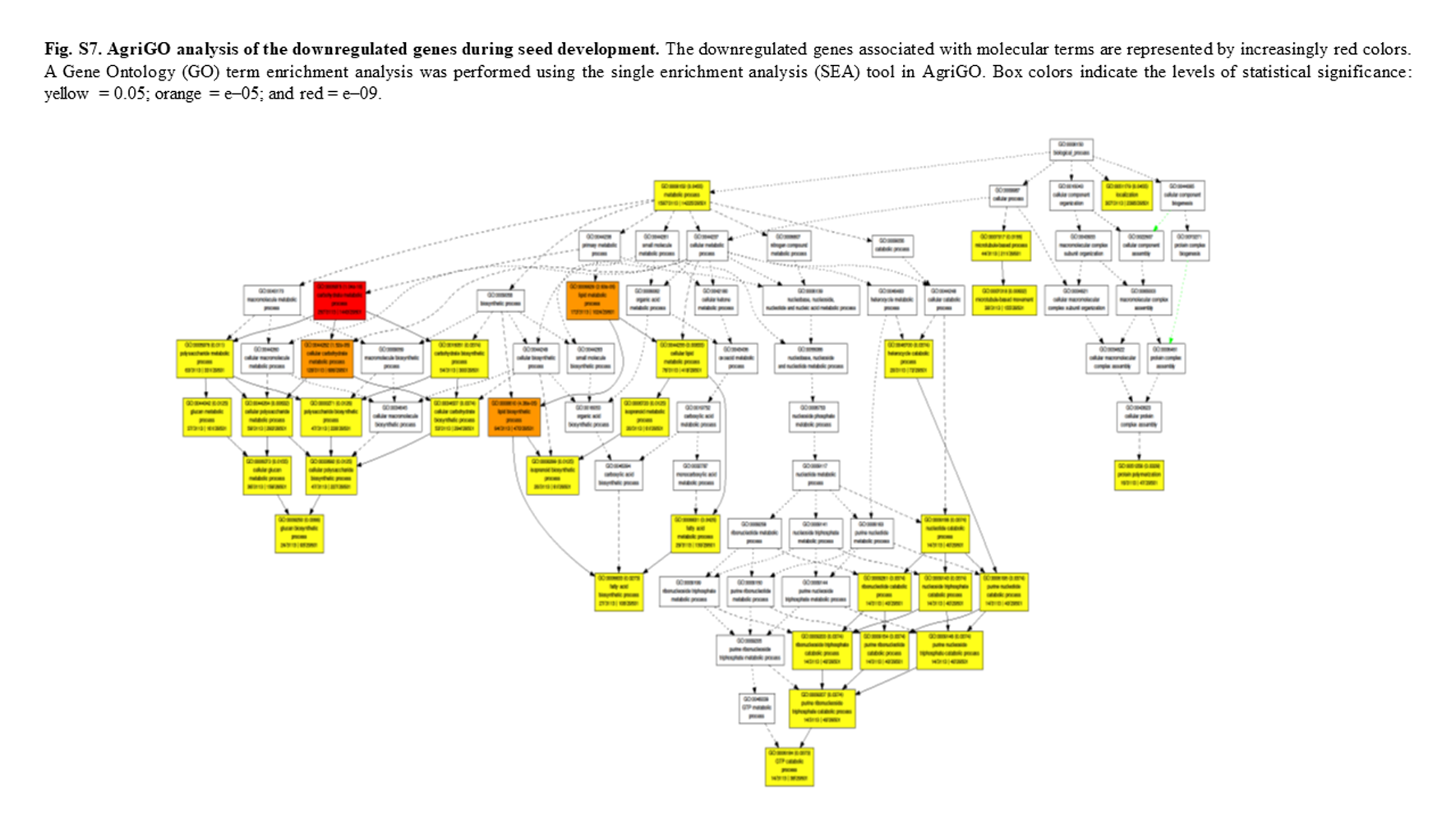

Supplement: Supplementary Figure 7 — AgriGO analysis of the downregulated genes during seed development. The downregulated genes associated with molecular terms are represented by increasingly red colors. A Gene Ontology (GO) term enrichment analysis was performed using the single enrichment analysis (SEA) tool in AgriGO. Box colors indicate the levels of statistical significance: yellow = 0.05; orange = e–05; and red = e–09. [file Image_7.tif]

C.

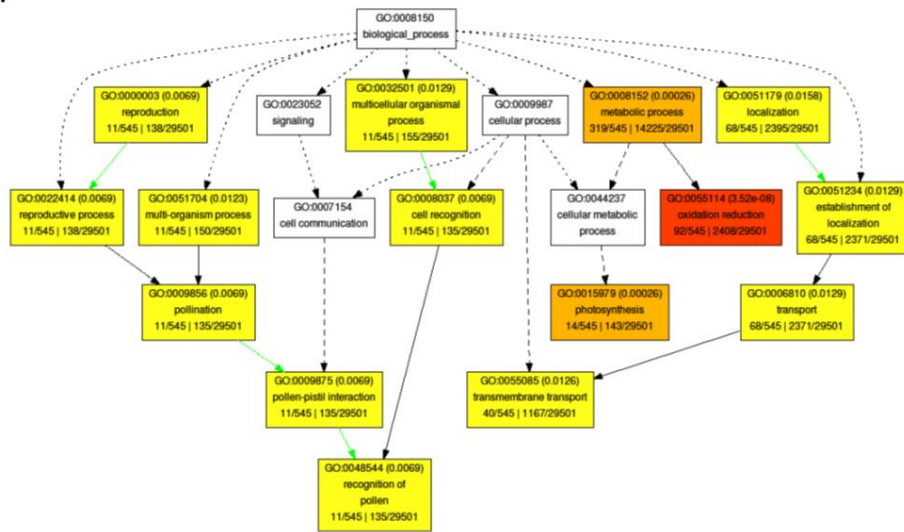

Supplement: Supplementary Figure 8 — AgriGO analysis of tissue-specific genes during seed development. Tissue-specific genes (A, leaf; B, root; and C, young_seed) associated with molecular terms are represented by increasingly red colors. A Gene Ontology (GO) term enrichment analysis was performed using the single enrichment analysis (SEA) tool in AgriGO. Box colors indicates the levels of statistical significance: yellow = 0.05; orange = e–05; and red = e–09. [file Image_8.pdf]
